# Draft Whole-Genome Sequence of a Klebsiella pneumoniae Strain Isolated from a Marmoset in Thailand

**DOI:** 10.1128/MRA.00503-21

**Published:** 2021-08-05

**Authors:** Komkiew Pinpimai, Wijit Bunlunara, Katriya Chankow, Rachod Tantilertcharoen, Pongthai Boonkam, Porntep Punnarak, Nopadon Pirarat

**Affiliations:** a Aquatic Resources Research Institute, Chulalongkorn University, Bangkok, Thailand; b Department of Pathology, Faculty of Veterinary Science, Chulalongkorn University, Bangkok, Thailand; c Veterinary Diagnostic Laboratory, Faculty of Veterinary Science, Chulalongkorn University, Bangkok, Thailand; Indiana University, Bloomington

## Abstract

Klebsiella pneumoniae is a Gram-negative bacterium that can cause infection in various kinds of animals and humans. Here, we report the genome sequence of K. pneumoniae isolated from a captive marmoset in Thailand.

## ANNOUNCEMENT

Klebsiella pneumoniae historically has been recognized as an opportunistic pathogen causing hospital-acquired infections (1). In the last few decades, K. pneumoniae has received more attention due to the increase in antibiotic resistance genes reported in classical strains and the emergence of hypervirulent strains that mainly cause primary liver abscesses and septicemia in young and healthy people (2, 3). K. pneumoniae infection is not limited to humans; there also are reports in a wide variety of animals (4–6). Here, we report the draft whole-genome sequence of K. pneumoniae isolated from a nonhuman primate, a marmoset (Callithrix jacchus), in Thailand.

A total of four captive marmosets (Callithrix jacchus) in a private house in Bangkok died suddenly without any previous clinical symptoms. All four carcasses were sent for necropsy at the Department of Pathology, Faculty of Veterinary Science, Chulalongkorn University, Thailand. K. pneumoniae isolates were recovered from the lung, liver, spleen, lymph nodes, and brain of all animals. Briefly, the tissues were inoculated onto blood agar and incubated at 37°C under aerobic conditions overnight. If a single colony type or a mixed culture with a predominance (>80%) of one colony type was seen, the colony was subcultured onto blood agar for further identification. All isolates were confirmed as K. pneumoniae using matrix-assisted laser desorption ionization–time of flight (MALDI-TOF) mass spectrometry (Bruker Daltonics, Billerica, MA). All isolates were of the hypermucoviscous phenotype (positive string test) (7). The K. pneumoniae isolate from the lung of marmoset number 1 was sequenced: the PureLink genomic DNA minikit (Invitrogen, CA, USA) was used to extract genome-quality DNA from a single colony cultured on blood agar, which was sent to Macrogen, Inc. (Seoul, South Korea), for whole-genome sequencing. A fragment library was prepared using a TruSeq Nano DNA library preparation kit (Illumina, Inc., San Diego, CA, USA). Paired-end reads (2× 100 bp) were obtained from a HiSeq instrument (Illumina, Inc.) and resulted in 10,642,888 reads. The overall coverage was estimated to be 98×. FastQC (8) was used to check the quality of the data. The sample was *de novo* assembled using SPAdes v3.14.1 (in “careful” mode) (9). The contigs produced using SPAdes were annotated with Prokka v1.14.5, with default parameters (10). The sequence type (ST) and serotype of the bacterial isolate were determined using the Bacterial Isolate Genome Sequence Database (BIGSdb) servers (http://bigsdb.pasteur.fr/klebsiella/klebsiella.html). The genome was 5,342,477 bp in size with a GC content of 57.25%. The genome was assembled into 82 contigs (*N*_50_, 138,887 bp). A total of 4,956 predicted protein-coding genes were identified, along with 3 rRNAs, 68 tRNAs, and 1 transfer-messenger RNA (tmRNA). The isolate was identified as the hypermucoviscous phenotype, serotype K2, and ST65.

The data from this study will provide information on the genome of K. pneumoniae isolated from marmoset and inform future research on the role of K. pneumoniae in animals.

### Data availability.

The draft genome sequence described here has been deposited in DDBJ/ENA/GenBank under accession number JAGLAL000000000, BioProject accession number PRJNA664586, and SRA accession number SRR12678423.
